# Potential of fermented herbal extracts to modulate digestion and gut microbiota during the weaner and fattening period on commercial pig farms

**DOI:** 10.3389/fvets.2025.1620045

**Published:** 2025-11-10

**Authors:** Barbara U. Metzler-Zebeli, Natalia Nöllenburg, Simone Koger, Katharina Schobersberger, Julia C. Vötterl, Christine Leeb

**Affiliations:** 1 Centre for Veterinary Systems Transformation and Sustainability, Clinical Department for Farm Animals and Food System Science, University of Veterinary Medicine Vienna, Vienna, Austria; 2 Department for Agricultural Sciences, BOKU University, Vienna, Austria; 3 Centre for Animal Nutrition and Welfare, Clinical Department for Farm Animals and Food System Science, University of Veterinary Medicine Vienna, Vienna, Austria

**Keywords:** fermentation, herbs, feces, microbiota, digestibility, farm, pig

## Abstract

Due to varying farm environments, the effect of feed additives on the gut microbiota and function in pigs may differ among farms. The present study aimed to evaluate the effect of a fermented herbal extract (FHE) on feed digestibility, fecal microbiota composition, and microbial metabolites under commercial production conditions on three pig farms throughout the weaner and fattening period. A total of 760 pigs across three farms were randomly allocated to one of two diets (control or 1% FHE) after weaning. On each farm, feces were collected from the same three barrows and three gilts per treatment at the weaner, mid, and the end of fattening period for microbiota, short-chain fatty acid (SCFA), and digestibility analyses. Total DNA from feces was extracted for 16S rRNA amplicon sequencing. Results were specific for farm, production stage (age), and sex. The FHE did not markedly affect the apparent total tract digestibility (ATTD) on Farms A and C (*p* > 0.05). On Farm B, the FHE improved the ATTD of crude ash by 11.4% in the mid-fattening period compared to the control (*p* < 0.05). On Farm B, the FHE increased (*p* = 0.041) the SCFA concentrations in feces of barrows (but not in gilts) compared to the control, but only in the mid-fattening period. On Farm C, FHE effects on SCFA fluctuated with age but were different compared to Farm B. The FHE increased (*p* < 0.05) or tended to increase (*p* < 0.10) species richness (observed features, Farms A and C) and diversity (Shannon, only Farm A) compared to the control in the weaner period. Likewise, more FHE-related changes in bacterial abundances were found in the weaner compared to the mid and end of fattening periods across farms, indicating that the FHE has more gut microbiota-modulating capabilities in younger pigs. The FHE-related changes in the bacterial composition were farm-specific and probably linked to the available fermentable substrate in the hindgut. Overall, results demonstrate the importance of investigating feed supplements like FHE on several farms and different production conditions to disentangle their gut physiological and microbial effects in weaner and fattening pigs.

## Introduction

Plant-based feed additives, also called botanicals, represent a wide group of compounds with different biological activities, including the stimulation of digestive secretions, antidiarrheal, and anti-inflammatory effects (1–3). Due to these biological activities, they are used as one nutritional strategy in pig production to sustain gut homeostasis, especially after weaning (1, 4, 5). Botanicals can be used in the form of the whole plant (fresh or dried) or as water and oil extracts (2). Because the intestinal absorption of biologically active compounds can be low, processing of the herbs or herbal extracts via fermentation can increase their bioavailability and simultaneously reduce potential toxicities (6–9, 11). Accordingly, Chinese fermented herbs have been shown to exert growth-promoting, immune-enhancing, antioxidative, and disease-resistant properties in weaned pigs (9, 11, 12). In fattening pigs, they may have the potential to reduce the fecal excretion of environmental pollutants such as ammonia (8). However, effects of botanicals reported in the literature on the porcine gut function and homeostasis vary, which has been associated with varying levels of bioactive compounds in the plant material or extracts (3). Another factor that may influence the effect of feed additives on the gut is the environment in which the pigs were raised (13). Studies conducted on commercial farms, partly under different hygienic and health conditions, demonstrated that the farm environment, including the housing system, management, and/or dietary composition, had a strong impact on the gut microbial composition from early in life (10, 13, 14). Despite this understanding, there is little scientific evidence on how the effect of a plant-based feed additive on the gut microbiota, their metabolites, and digestion varies when fed to weaned and fattening pigs on different farms. Therefore, studying a feed additive under production conditions on more than one farm can help us understand its effectiveness. Furthermore, gut microbiota responses to treatments can be different between sexes (15), which should be considered more when investigating the effects of feed additives.

The objective of this study was to investigate the effect of a fermented herbal extract (FHE) consisting of 12 herbs on the feed digestibility, fecal microbiota composition, and microbial metabolites in gilts and barrows on three commercial pig farms throughout the weaner and fattening period. In doing so, we could assess the FHE effects when pigs were raised in different environments and fed different diets. In order to follow the pigs from weaning to slaughter, we collected feces to determine the FHE effects on the gut microbiota and digestibility. We hypothesized that the effect of the applied FHE on the gut microbiota would show certain farm- and age-specific dynamics, but that the effects would be similarly directed across farms. Moreover, we hypothesized that FHE effects on the gut microbiota and nutrient digestibility would be similarly directed in gilts and barrows. We applied a commercially available FHE product to test our hypotheses, which was produced under standardized procedures.

## Materials and methods

### Animals and housing

The experiment was conducted between June 2022 and July 2023 in three replicate batches on three farms that were located in the region of Upper Austria (Austria). The three farms produce for the same regional Austrian meat company and welfare label^1^ and were selected to have relatively uniform housing systems with 400 to 450 weaning-fattening places working in a three-week cycle (Supplementary Table S1). The experimental period lasted throughout the weaning-fattening period from weaning to slaughter. Per farm, each replicate batch was performed at a different time of the year; each covered two seasons.

Pigs were crossbreeds [Landrace × Large White (sow) and Piétrain (boar)], weaned on average at 28 days of age (range 23–33 days of life) and slaughtered after 6 to 7 months (on average 191 days of life). Detailed information on the housing system of the individual farms can be found in Supplementary Table S1. On each of the three farms, weaned pigs were randomly allotted to the FHE and control treatments with similar numbers of barrows and gilts and uniform distribution of litters. In the weaner period, there were two pens that received the diet with the added FHE (*n* = 20–25 pigs/pen) and two control pens (*n* 20–25 pigs/pen) in the same room next to each other (Supplementary Table S1). Pigs stayed in the weaner pens until they reached an average body weight of 36 kg (mean ± SD: 35.7 ± 6.89 kg) on Farms B and C. Farm A transferred the pigs earlier to the fattening units after 5 weeks of weaning with an average body weight of 21 kg (mean ± SD: 21.0 ± 1.86 kg). For the fattening period, pigs from the two pens per treatment were mixed into one pen per treatment. Farmers were asked to provide the same conditions and type of manipulable materials, usually consisting of chains, wooden blocks, hay, or straw, for all pigs.

### Diets and feeding

The feeding management and dietary compositions for the weaner and fattening periods corresponded to the regular rations fed on the respective farm. The rations were formulated by the feed consultant of the respective farm and met or surpassed the recommendations for nutrient requirements of pigs in the respective production stage (16). Weaned pigs received prestarter, starter I, and starter II diets on Farms A and C, whereas on Farm B, only one starter diet was fed throughout the weaner period. In the fattening period, only one diet was fed to the pigs on all three farms. The diets were mixed by the farmers. The diets fed close to the fecal samplings are shown in Supplementary Table S2. The diets contained ingredients from regional production (grains mostly produced on the farm and protein feedstuffs from the region) and were genetically modified organism-free. The FHE was a commercial product (Multikraft, Pichl bei Wels, Austria) that was produced under standardized conditions. The production conditions and chemical composition are both proprietary. It consisted of a mixture of sugar cane molasses, lactose powder, and different plant juices (i.e., birch leaves, raspberry leaves, caraway, yarrow, fennel, thyme, rosemary, peppermint, marshmallow root, anise, goldenrod, and milk thistle seed). This mixture was fermented under the influence of different strains of *Lactobacillaceae* (*Lacticaseibacillus casei, Limosilactobacillus fermentum, Lactiplantibacillus plantarum,* and *Lacticaseibacillus rhamnosus*) and yeast (*Saccharomyces cerevisiae*). The FHE was supplemented at a concentration of 1% per 88% dry matter directly before feeding. This corresponded to a daily FHE intake of 5 and 30 ml with an assumed feed intake of 500 g in the mid-weaner period and 3 kg in the mid-fattening period, respectively. During the experiment, the FHE tank was connected to the feed pre-mixing container via a hose. As soon as the dry feed mixture was poured into the pre-mixing container, the liquid FHE was added to the mixture. The homogeneously mixed feed was then forwarded to the respective troughs via a tubular track system (spotmix or tubular chain). The control treatment received the basal diet without the FHE. Information about the feeding can be found in Supplementary Table S1. Water was freely available to the pigs.

### Collection of feed and fecal samples

The experiment was blinded to the observer, and only the farmers knew the allocation of the pigs to the FHE and control treatments. Feed and fecal samples were collected during three visits at each farm. At these visits, the health of the pigs was also visually assessed. Furthermore, farmers were asked to report all medical treatments, deaths, and other incidents. Samples from the various feeds were collected at each farm at the same time and stored at −20 °C. The fecal samples were collected from the same three barrows and three gilts per treatment, with two barrows and one gilt in one pen and vice versa in the second pen. Only results from the selected animals are presented in this paper. The selected pigs were identified with special markings on their ear tags. The first sample collection took place at the end of the weaner period (Farm B: 13.0 ± 0.51; Farm C: 11.5 ± 0.51 (SD) weeks of age), the second in the mid of the fattening period (Farm B: 19.5 ± 1.53; Farm C: 18.5 ± 0.51 (SD) weeks of age), and the third collection was at the end of the fattening period, usually 1 day before slaughter (Farm B: 26.0 ± 0 (SD); Farm C: 27.5 ± 0.51 (SD) weeks of age). Pigs from Farm A were sampled earlier in the weaner period (8.7 ± 0.48 (SD) week of age) as they were transferred to the fattening period at a younger age compared to the pigs on Farms B and C. Fecal samples were collected on 1 day per sampling time point. To obtain fecal samples, each animal was isolated from the pen in a separate space (usually the corridor). The pig was observed until defecation occurred. Immediately thereafter, material from the inner part of the feces was taken, placed on ice, and later homogenized for aliquoting. One aliquot was filled into DNA-free tubes for microbiota analysis, and the bigger aliquot into plastic containers for digestibility analysis. Both aliquots were transferred to the laboratory and stored at −20°C. On Farm A, there were problems with the dosing system in the fattening barn. Due to this reason, the fattening period from Farm A was excluded from analysis. From Farms B and C, feces collected at the end of the weaner period and in the mid and at the end of the fattening period were chemically and microbiologically analyzed from two replicate batches only, as too many selected pigs were medically treated from the third replicate batch at both farms. Consequently, there were six barrows and six gilts per treatment that were included on Farms B and C over the two replicate batches for laboratory analysis. We performed *a priori* power analysis (17) using the PROC Power of SAS (version 9.4; SAS Institute, Cary, NC), which was based on data from previous studies on the fecal microbiota (18) to ensure that several biological replicates of 6 would allow us to reject the null hypothesis if this was false.

### Proximate nutrient analysis in feed and feces

Feed samples that were collected at the farm visits for the fecal samplings were sent for proximate nutrient analysis to a commercial feed laboratory (Feed Laboratory Rosenau, Haag, Austria; Supplementary Table S2). This included the starter diets from all farms and the fattening diets on Farms B and C. Dry matter (DM), crude ash (CA), crude protein (CP), ether extract, crude fiber, total starch, and sugar were measured according to the methods described by Naumann and Bassler (19). For the DM (method 3.1), feed samples were oven-dried at 103 °C for 4 h. For CA (method 3.5), the feed was incinerated at 580 °C for 4 h (19). The ether extract (method 5.1.1) was analyzed by solvent extraction with petroleum ether. The total N content was determined according to DUMAS and converted into CP by multiplying the N content by 6.25 (method 4.1.2). The nitrogen-free extract fraction was calculated by subtracting crude ash, crude protein, crude fiber, and ether extract from the DM (19). Total starch was determined using the polarimetric method (method 7.2.1). One subsample of the feces from each pig at the three sampling time points was freeze-dried before they were ground to pass a 1-mm sieve and analyzed for DM, CA, and CP (19). Furthermore, fecal and feed samples were analyzed for acid-insoluble ash (method 8.2) (19) as an indigestible marker. The apparent total tract digestibility (ATTD) of DM, CA, and CP was calculated using the following equation:


$$\text{ATTD}\;(\%)=100-[(\frac{\text{Nutrient feces}}{\text{Nutrient feed}})\times (\frac{\text{Acid insoluble}\;\mathrm{ash}\;\text{feed}\;}{\text{Acid insoluble}\;\mathrm{ash}\;\text{feces}})\times 100]$$

### DNA extraction and sequencing

From 250 mg of feces, total DNA was extracted using the DNeasy PowerSoil Pro Kit (Qiagen, Hilden, Germany) with the same modifications, including a heating step and mechanical lysis as described in Lerch et al. (14). The concentration of DNA in each extract was measured with a Qubit fluorometer (Qubit 4 Fluorometer, Thermo Fisher Scientific Inc., Waltham, MA, USA) using the Qubit 1XdsDNA HS Assay Kit. Targeted 16S rRNA gene sequencing (V3-V4 hypervariable region) was performed in an external laboratory (Microsynth AG, Balgach, Switzerland). Aliquots of the DNA extracts were sent for library preparation (NEBNext Ultra II DNA Library Prep Kit, Illumina, San Diego, CA, USA). The 16S rRNA gene amplicon was amplified using primers 341F-ill (5′-CCTACGGGNGGCWGCAG-3′) and 805R-ill (5′-GACTACHVGGGTATCTAATCC-3′) (20). Equimolar pools of samples were sequenced to generate 250 bp paired-end raw reads in the Novaseq 6000 platform (Illumina). Demultiplexing and trimming of the raw sequences were performed by Microsynth.

Trimmed reads for the 16S rRNA amplicons were processed, denoised, and classified using the Divisive Amplicon Denoising Algorithm 2 (version 1.26.0) in R Studio (version 1.4.1106) (21). The forward and reverse read quality profiles were separately examined. To account for the decrease in quality score of the subsequent nucleotides, the total length of forward and reverse reads was truncated to 220 nucleotides with a maximum error rate of 5 for both forward and reverse reads (truncQ = 5) using the ‘filterAndTrim’ function. Furthermore, this function was used to pre-filter sequences to remove reads with ambiguous bases. Amplicon sequence variants were inferred after de-replication of the filtered data and estimation of error rates (21). The inferred forward and reverse sequences were then merged, with paired sequences that did not perfectly match removed to control for residual errors, and a sequence table was constructed. The ‘removeBimeraDenovo ()’ function was used to remove chimeras, and taxonomy was assigned using the SILVA 138.1 ribosomal RNA database (22). The raw sequence counts from the taxa table at the genus level were collapsed and compositionally normalized such that each sample summed to 1. The relative abundances at the genus rank were statistically analyzed as described below. Alpha-diversity (Shannon, Simpson, observed features) analysis was performed using phyloseq (version 1.42.0). For beta-diversity analysis, statistical assessment of dissimilarity matrices (Bray-Curtis) was performed using the ‘adonis2’ function in the R package ‘vegan’ (version 2.6.4) (23). The permutational multivariate analysis of variance (PERMANOVA) was used on the Bray-Curtis distance matrices to assess the dissimilarities among the bacterial communities in feces on the various sampling days, farms, sex, and treatment. The statistical significance was determined after 999 random permutations. The two-dimensional non-metric multidimensional scaling (NMDS) ordination plots were generated using the ‘metaMDS’ function. The ggplot2 package was used to visualize the clustering of bacterial communities among sampling days, farms, sex, and treatments.

### Short-chain fatty acid analysis in feces

In the second subsample of feces from each pig, concentrations of short-chain fatty acids (SCFA), including acetate, propionate, butyrate, iso-butyrate, valerate, iso-valerate, caproate, and heptanoate, were determined using gas chromatography after extraction with 25% phosphoric acid and addition of 4-methylvaleric acid as an internal standard (Sigma-Aldrich, St. Louis, MO, USA). Homogenized sample solutions were centrifuged (20,000 × *g* for 20 min). If necessary, samples were centrifuged several times, until a clear supernatant was obtained, which was used to measure SCFA on the GC-2010 Plus Capillary gas chromatograph (Shimadzu Corp., Kyoto, Japan) using a 30 m × 0.53 mm × 0.5 μm capillary column (Trace TR Wax, Thermo Fisher Scientific, Waltham, MA, USA) and helium as carrier gas. The gas chromatograph was equipped with an autosampler and injector (AOC-20s Auto Sampler; AOC-20i Auto-Injector, Shimadzu Corp., Kyoto, Japan) and a flame-ionization detector (FID-2010 Plus, Shimadzu Corp., Kyoto, Japan). The SCFA were expressed as concentrations.

### Statistical analysis

The residuals of the data for fecal DM, amounts of CP and CA in feces, ATTD coefficients, fecal SCFA, bacterial taxonomy, and alpha-diversity were first tested for normal distribution using the Shapiro–Wilk test with the UNIVARIATE procedure in SAS (version 9.4; SAS Institute, Inc., Cary, NC, USA). If the residuals were not normally distributed, data were transformed using the Box-Cox method and the Transreg procedure in SAS. Next, the aforementioned datasets were subjected to ANOVA using the MIXED procedure in SAS. For the ANOVA of the bacterial genera, relative abundances > 0.5% of all reads were analyzed. Because the age of pigs at the fecal samplings differed at the three farms, the various parameters were analyzed by farm and production stage. Beta-diversity analysis (PERMANOVA) of the microbiota supported the validity of analyzing the data separately per farm and age. From Farm A, as mentioned above, only fecal samples from the weaner period were analyzed. The fixed effects included replicate batch, sex, treatment, and their interactions. The random effect was replicate batch, and the experimental unit was pig nested within pen. Degrees of freedom were approximated by the Kenward-Rogers method (ddfm = kr). Data were reported as the least-square means ± standard errors of the mean (SEM). Multiple pairwise comparisons among least-square means were performed using the pdiff statement. For the bacterial taxonomy (genus level), the Bonferroni correction was applied to adjust the raw *p* values for the relative abundances. A significant difference was defined at *p* ≤ 0.05 and trends at 0.05 < *p* ≤ 0.10.

## Results

### Dietary composition

The chemical composition of the feeds at the time of fecal sampling is presented in Supplementary Table S2. Briefly, the CP content of the starter feed was 18.0% (DM basis) on Farm A, whereas it was 17.8 and 17.9% on a DM basis in the starter feed (starter II) on Farms B and C, respectively. The CP content in the fattening feed on Farms B and C amounted to 17.5 and 18.0% (DM basis), respectively. The crude fiber contents ranged from 3.9 to 6% (DM basis) in the different feeds and farms.

### Fecal composition and ATTD

On Farm A (Table 1), the fecal composition and ATTD of DM, CP, and CA were similar between gilts and barrows in the weaner period. By contrast, sex effects occurred for the fecal composition in the weaner and fattening periods on Farms B and C. Gilts tended (*p* = 0.098) to contain more CA in their feces compared to barrows in the weaner period on Farm B. Also, gilts had a 7.3% lower fecal CP content compared to barrows in the mid-fattening period on Farm B (*p* = 0.047). At the end of the fattening period, the ATTD of DM and CP were 3.9 and 2.4% higher in gilts compared to males, respectively, on Farm B (*p* < 0.05). Similarly, on Farm C, gilts had a higher ATTD of CA in the weaner period and of DM, CP, and CA in the mid-fattening period compared to barrows (*p* < 0.05).

**Table 1:** Differences in the apparent total tract digestibility and fecal contents of dry matter, protein, and ash in pigs fed the control or FHE diet in the weaner and fattening period at the three farms.

Item	Control diet		FHE diet		SEM	*p*-value		
	M	F	M	F		Sex	Treatment	Treatment × sex
Farm A								
Weaner period								
Feces DM (%)	79.2	77.7	77.9	78.1	0.99	0.766	0.084	0.814
Feces CP (%)	85.8	84.5	84.3	85.0	0.80	0.353	0.225	0.393
Feces CA (%)	57.7	55.6	59.7	57.7	2.16	0.881	0.264	0.298
ATTD of DM (%)	24.1	24.7	22.2	22.3	1.20	0.505	0.638	0.408
ATTD of CP (%)	21.6	25.6	26.3	26.5	2.25	0.743	0.538	0.227
ATTD of CA (%)	12.8	12.1	11.3	12.1	0.69	0.357	0.348	0.974
Farm B								
Weaner period								
Feces DM (%)	82.9	83.7	82.0	81.9	2.15	0.457	0.752	0.130
Feces CP (%)	87.3	88.6	88.2	90.8	1.73	0.141	0.890	0.800
Feces CA (%)	65.6	65.9	61.6	55.0	4.17	0.098	0.045	0.186
ATTD of DM (%)	20.9	21.5	21.8	20.1	0.75	0.852	0.540	0.838
ATTD of CP (%)	21.5	16.3	21.9	14.8	3.82	0.302	0.391	0.735
ATTD of CA (%)	11.4	11.7	12.0	14.4	0.75	0.461	0.093	0.424
Mid-fattening period								
Feces DM (%)	82.0	81.1	82.0	83.5	0.89	0.105	0.220	0.278
Feces CP (%)	86.5	85.5	86.5	86.1	0.77	0.047	0.295	0.150
Feces CA (%)	42.0	47.0	50.0	49.1	2.11	0.653	0.708	0.048
ATTD of DM (%)	23.1	20.2	23.2	22.6	1.00	0.759	0.211	0.192
ATTD of CP (%)	21.2	20.7	21.5	18.8	0.72	0.362	0.724	0.704
ATTD of CA (%)	14.1	12.1	12.2	13.5	0.78	0.340	0.027	0.185
End of fattening period								
Feces DM (%)	75.9	77.7	74.0	78.0	1.14	0.447	0.505	0.208
Feces CP (%)	81.5	81.5	78.8	82.6	0.84	0.516	0.972	0.152
Feces CA (%)	22.4	21.6	17.7	24.8	3.90	0.487	0.723	0.402
ATTD of DM (%)	20.7	21.6	21.7	18.3	1.61	0.027	0.494	0.378
ATTD of CP (%)	21.1	19.4	19.9	20.5	0.77	0.043	0.322	0.039
ATTD of CA (%)	14.0	15.1	14.9	14.8	0.74	0.449	0.850	0.328
Farm C								
Weaner period								
Feces DM (%)	79.3	81.5	76.4	79.6	1.83	0.495	0.057	0.554
Feces CP (%)	84.4	86.1	82.3	84.2	2.00	0.394	0.812	0.866
Feces CA (%)	47.8	57.5	43.6	52.0	3.91	0.554	0.733	0.422
ATTD of DM (%)	20.1	20.2	21.9	23.5	1.24	0.138	0.261	0.781
ATTD of CP (%)	24.2	23.0	23.7	22.9	1.16	0.341	0.344	0.958
ATTD of CA (%)	13.8	12.8	13.0	13.2	0.70	0.030	0.278	0.867
Mid-fattening period								
Feces DM (%)	61.3	73.6	64.1	74.5	2.74	0.184	0.170	0.738
Feces CP (%)	67.9	76.9	72.2	79.5	2.58	0.224	0.104	0.782
Feces CA (%)	6.6	23.4	12.2	33.2	4.95	0.139	0.478	0.144
ATTD of DM (%)	16.7	19.8	14.8	16.7	1.74	0.002	0.509	0.724
ATTD of CP (%)	19.9	21.1	21.5	22.2	0.74	0.012	0.229	0.758
ATTD of CA (%)	13.5	18.6	14.8	14.9	1.64	0.003	0.156	0.683
End of weaner period								
Feces DM (%)	74.6	71.3	75.1	74.6	1.97	0.881	0.263	0.696
Feces CP (%)	77.2	71.7	78.1	76.9	2.18	0.231	0.101	0.695
Feces CA (%)	9.8	20.6	17.3	35.0	6.94	0.050	0.641	0.705
ATTD of DM (%)	17.5	17.8	19.7	18.9	1.41	0.354	0.341	0.490
ATTD of CP (%)	19.6	18.4	20.6	20.0	0.73	0.146	0.180	0.353
ATTD of CA (%)	20.5	16.7	22.4	16.9	2.19	0.067	0.163	0.624

Values are LS means ± SEM. ATTD, apparent total tract digestibility; CA, crude ash; CP, crude proteins; DM, dry matter; F, gilt; FHE, fermented herbal extract; M, barrow.

The FHE tended (*p* = 0.079) to lower the fecal DM content by 8.9% compared to pigs fed the control diet on Farm A. Moreover, on Farm B, the FHE addition tended (*p* = 0.095) to reduce the ATTD of CA by 11.3% while increasing the fecal CA concentration by 14.0% in the weaner period compared to the control (*p* = 0.045). When the pigs got older, the FHE addition enhanced the ATTD of CA by 11.5% in the mid-fattening period compared to the control pigs on Farm B (*p* = 0.027). Furthermore, depending on sex, FHE increased or decreased fecal CA as indicated by the dietary treatment and sex interaction (*p* = 0.048). Except for a trend for a higher DM content in feces for pigs receiving the FHE diet (*p* = 0.057) at the end of the weaner period, no differences in the ATTD and contents of nutrients in feces between the two treatments were observed on Farm C.

### Fecal microbiota structure and genera composition

**Figure 1:**
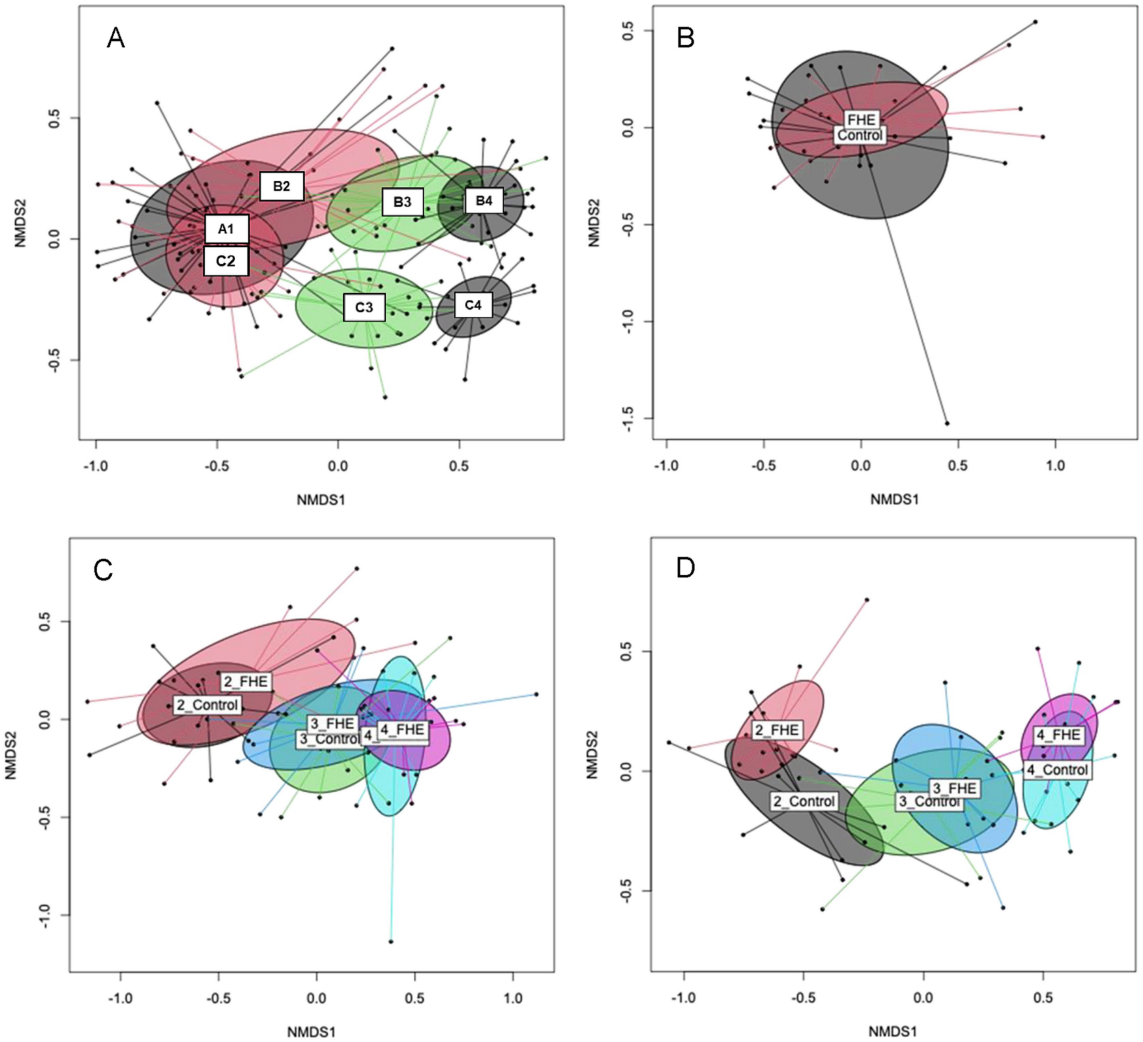
Non-metric multidimensional scaling (NMDS) plot of pairwise Bray–Curtis dissimilarities in the bacterial communities in feces for production stages of all farms **(A)**, treatments (FHE, Control) in weaner period (A1) on Farm A **(B)**, treatment groups at the different production stages [weaner (B2), mid- (B3), end of fattening (B4)] on Farm B **(C)** and treatments at the different production stages [weaner (C2), mid- (C3), end of fattening (C4)] on Farm C **(D)**. Ellipses represent the standard deviation. 2_FHE, weaner period and fermented herbal extract diet; 2_Control, weaner period and control diet; 3_FHE, weaner period and fermented herbal extract diet; 3_Control, weaner period and control diet; 4_FHE, weaner period and fermented herbal extract diet; 4_Control, weaner period and control diet.

The beta-diversity analysis showed that the farm and production stage affected the fecal bacterial communities, as illustrated in NMDS plots (Figure 1). The bacterial communities in the feces of pigs from Farms A, B, and C showed a certain overlap in the weaner period, indicating similarities at this age. By contrast, in the mid and at the end of the fattening period, bacterial communities in the feces of pigs from Farms B and C clustered separately. The PERMANOVA further indicated that sex did not influence the overall bacterial community structure in pigs’ feces at the three farms (Supplementary Table S3). For Farms B and C, the PERMANOVA showed an interactive effect for production stage and diet (*p* = 0.001). Moreover, total bacterial gene copy numbers showed small differences of only 0.1- to 0.2-log units among sexes and treatments (Supplementary Table S4).

The ANOVA demonstrated that sex tended to influence the species richness (observed features) and diversity (Shannon and Simpson) at the end of the fattening period, but only on Farm C, where gilts tended to have lower indices than barrows (*p* < 0.10; Table 2). The ANOVA further indicated FHE effects on the alpha-diversity and relative abundances of bacterial genera. On Farm A, the FHE increased the species richness (observed features; *p* < 0.046) and diversity (Shannon; *p* < 0.084) in pigs in the mid-weaner period. Likewise, on Farm C, the FHE tended (*p* = 0.093) to increase the species richness (observed features) by 21.7 species at the end of the weaner period, but not on Farm B.

**Table 2:** Alpha-diversity of bacterial community in feces of pigs fed the control or FHE diet in the weaner and fattening period at the three farms.

Item	Control diet		FHE diet		SEM	*p*-value		
	M	F	M	F		Sex	Treatment	Treatment × sex
Farm A								
Weaner period								
Observed features	59.7	66.2	94.8	94.7	15.28	0.836	0.046	0.827
Shannon	3.28	3.51	3.69	3.66	0.156	0.520	0.084	0.420
Simpson	0.94	0.96	0.96	0.96	0.010	0.342	0.205	0.324
Farm B								
Weaner period								
Observed features	77.0	83.2	88.5	101	9.73	0.350	0.148	0.748
Shannon	3.68	3.77	3.64	3.85	0.104	0.174	0.888	0.550
Simpson	0.96	0.97	0.96	0.97	0.004	0.226	0.981	0.562
Mid-fattening period								
Observed features	107	103	102	89.0	15.85	0.616	0.539	0.784
Shannon	3.74	3.77	3.92	3.61	0.192	0.465	0.945	0.391
Simpson	0.96	0.96	0.97	0.96	0.009	0.402	0.822	0.434
End of fattening period								
Observed features	97.8	125	117	103	13.46	0.626	0.922	0.140
Shannon	3.71	4.02	3.99	3.82	0.157	0.637	0.799	0.147
Simpson	0.96	0.97	0.97	0.96	0.007	0.837	0.793	0.124
Farm C								
Weaner period								
Observed features	90.7	96.7	127	104	12.31	0.478	0.093	0.241
Shannon	3.75	3.81	3.95	3.87	0.085	0.912	0.144	0.378
Simpson	0.97	0.97	0.97	0.97	0.003	0.907	0.139	0.485
Mid-fattening period								
Observed features	140	122	135	130	23.00	0.645	0.952	0.780
Shannon	3.98	3.93	3.81	3.95	0.132	0.744	0.570	0.452
Simpson	0.97	0.97	0.96	0.97	0.005	0.746	0.409	0.518
End of fattening period								
Observed features	170	128	177	141	19.81	0.072	0.619	0.884
Shannon	4.21	3.92	4.21	4.07	0.119	0.099	0.531	0.553
Simpson	0.98	0.97	0.98	0.97	0.003	0.060	0.315	0.428

Values are LS means ± SEM. F, gilt; FHE, fermented herbal extract; M, barrow.

**Figure 2:**
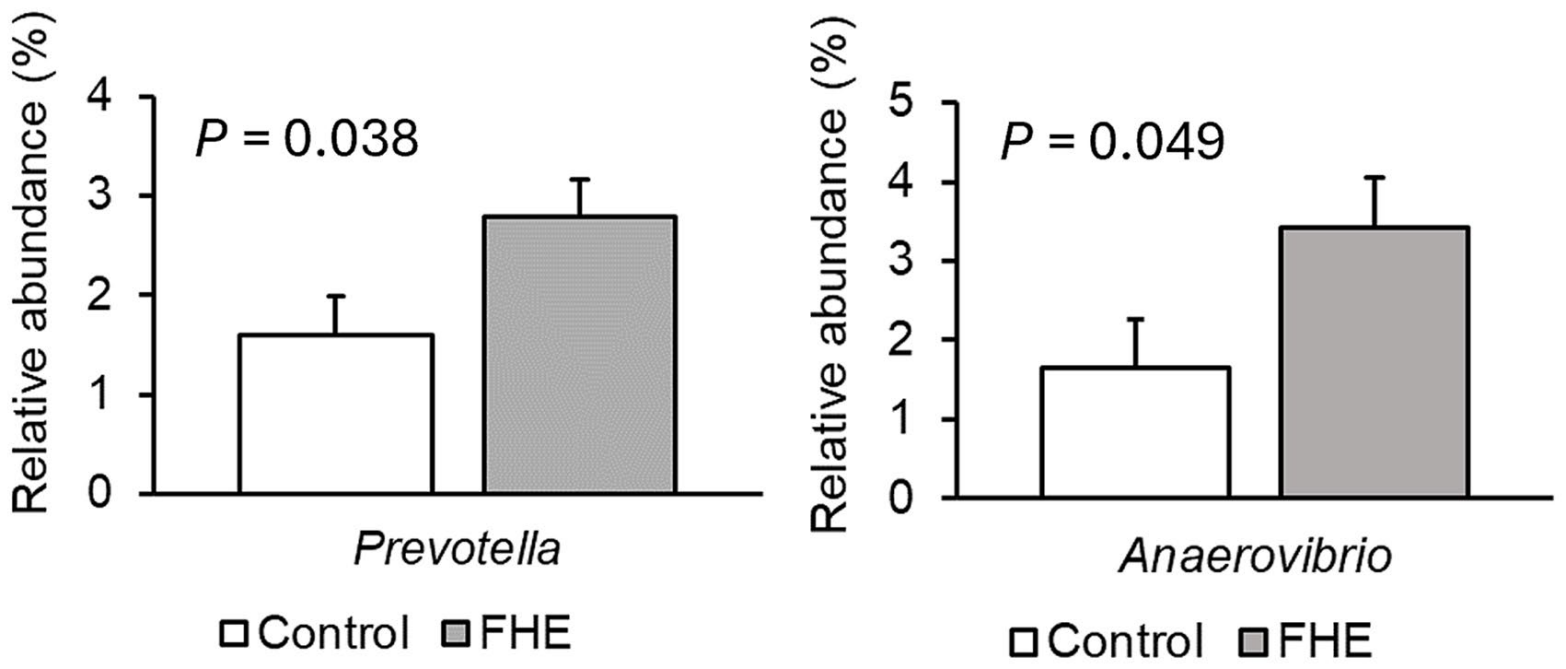
Differences in relative abundances (% of all reads) of bacterial genera in feces of pigs fed the control diet (white bar) or diet with added fermented herbal extract (grey bar) in the weaner period on Farm A. Values are least-squares means ± standard error of the mean.

**Figure 3:**
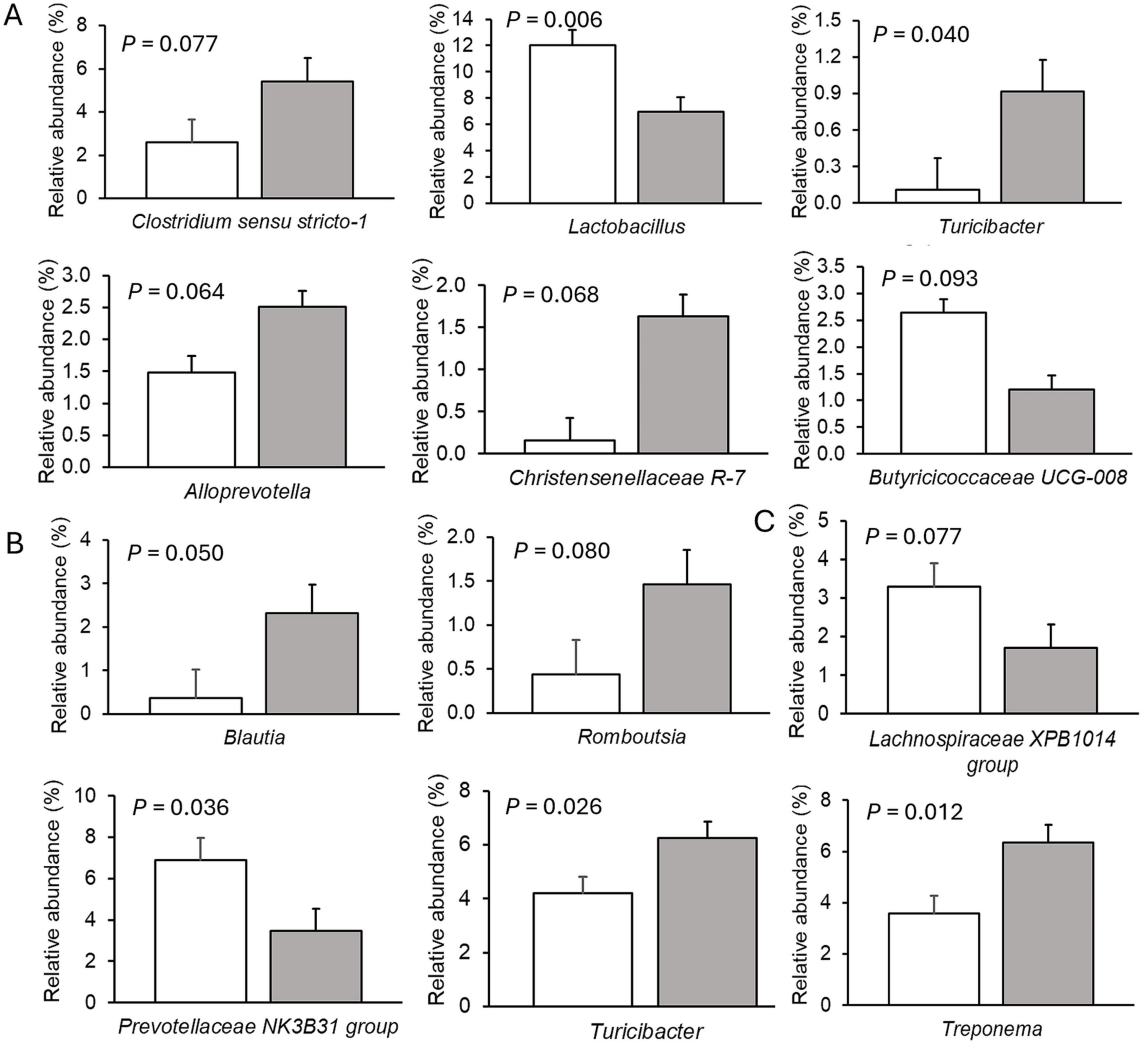
Differences in relative abundances (% of all reads) of bacterial genera in feces of pigs fed the control diet (white bar) or diet with added fermented herbal extract (grey bar) on Farm B. **(A)** Weaner period, **(B)** mid-fattening period, and **(C)** end of fattening period. Values are least-squares means ± standard error of the mean.

**Figure 4:**
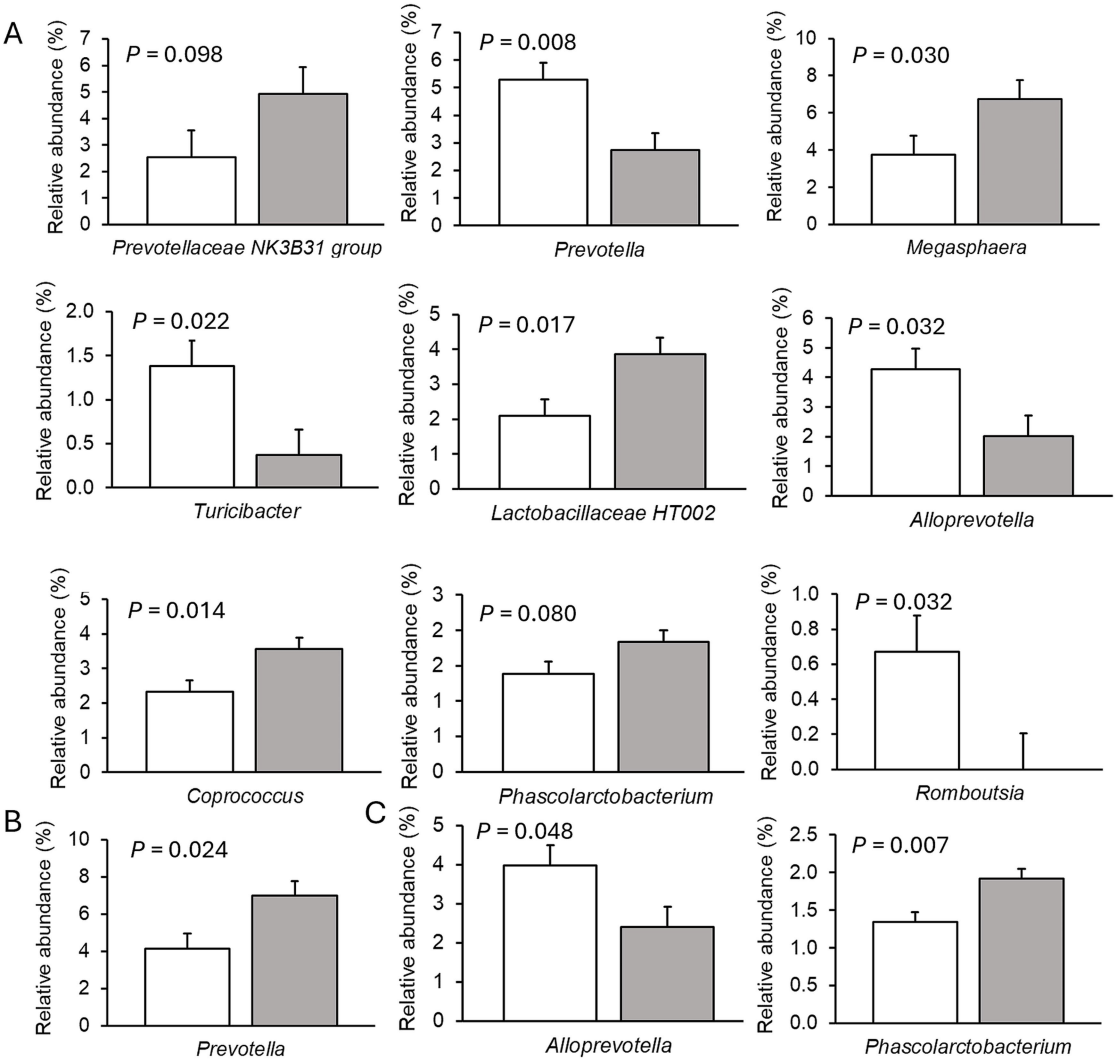
Differences in relative abundances (% of all reads) of bacterial genera in feces of pigs fed the control diet (white bar) or diet with added fermented herbal extract (grey bar) on Farm C. **(A)** Weaner period, **(B)** mid-fattening period, and **(C)** end of fattening period. Values are least-squares means ± standard error of the mean.

Only very few differences in the relative abundances of bacterial genera existed between sexes. Therefore, only dietary effects on the relative abundances of genera are presented (Figures 2–4; Supplementary Tables S5–S7). The FHE effects differed for the three farms and for the three sampling time points in the weaner and fattening period. On Farm A (Figure 2; Supplementary Table S5), the FHE increased the relative abundance of *Prevotella* and *Anaerovibrio* by 7.4 and 10.9%, respectively, compared to the control diet, in the mid-weaner period (*p* < 0.05). On Farm B (Figure 3; Supplementary Table S6), the FHE decreased (*p* = 0.006) *Lactobacillus* from 12 to 7% relative abundance but simultaneously increased other genera such as *Clostridium* sensu stricto 1 (*p* = 0.077), *Turicibacter* (*p* = 0.040), *Alloprevotella* (*p* = 0.064), and *Christensenellaceae* R-7 group (*p* = 0.068) from 0.7- to 9-fold in feces compared to the control diet in the weaner period. In the mid-fattening period, the effects of FHE were different. They were limited to an increase in *Blautia* and *Romboutsia* by 5.4- and 2.3-fold, respectively, while decreasing an unclassified *Lachnospiraceae* genus (*Lachnospiraceae* XPB1014 group) by 0.5-fold compared to the control diet (*p* < 0.10) on Farm B. At the end of the fattening period, the FHE decreased the relative abundance of the *Prevotellaceae* NK3B31 group by 0.5-fold while increasing the abundance of *Turicibacter* and *Treponema* by 0.5- and 0.8-fold, respectively, compared to the control diet on Farm B (*p* < 0.05). On Farm C (Figure 4; Supplementary Table S7), the FHE affected (*p* < 0.05) or tended (*p* < 0.10) to affect nine genera. This included an increase in the relative abundance of the *Prevotellaceae* NK3B31 group, *Megasphaera*, *Lactobacillaceae* HT002, *Coprococcus*, and *Phascolarctobacterium* by 0.9-, 0.8-, 0.8-, 0.5-, and 0.3-fold, respectively, and a decrease in *Prevotella*, *Turicibacter*, *Alloprevotella,* and *Romboutsia* by 0.5- to 10-fold compared to the control diet at the end of the weaner period. In the mid-fattening period, the FHE increased (*p* = 0.024) the relative abundance of one genus, *Prevotella*, by 0.7-fold, whereas at the end of the fattening period, two genera responded to the FHE (decrease in *Alloprevotella* and increase in *Romboutsia* by 0.4-fold each; *p* < 0.05) compared to the control diet on Farm C.

**Figure 5:**
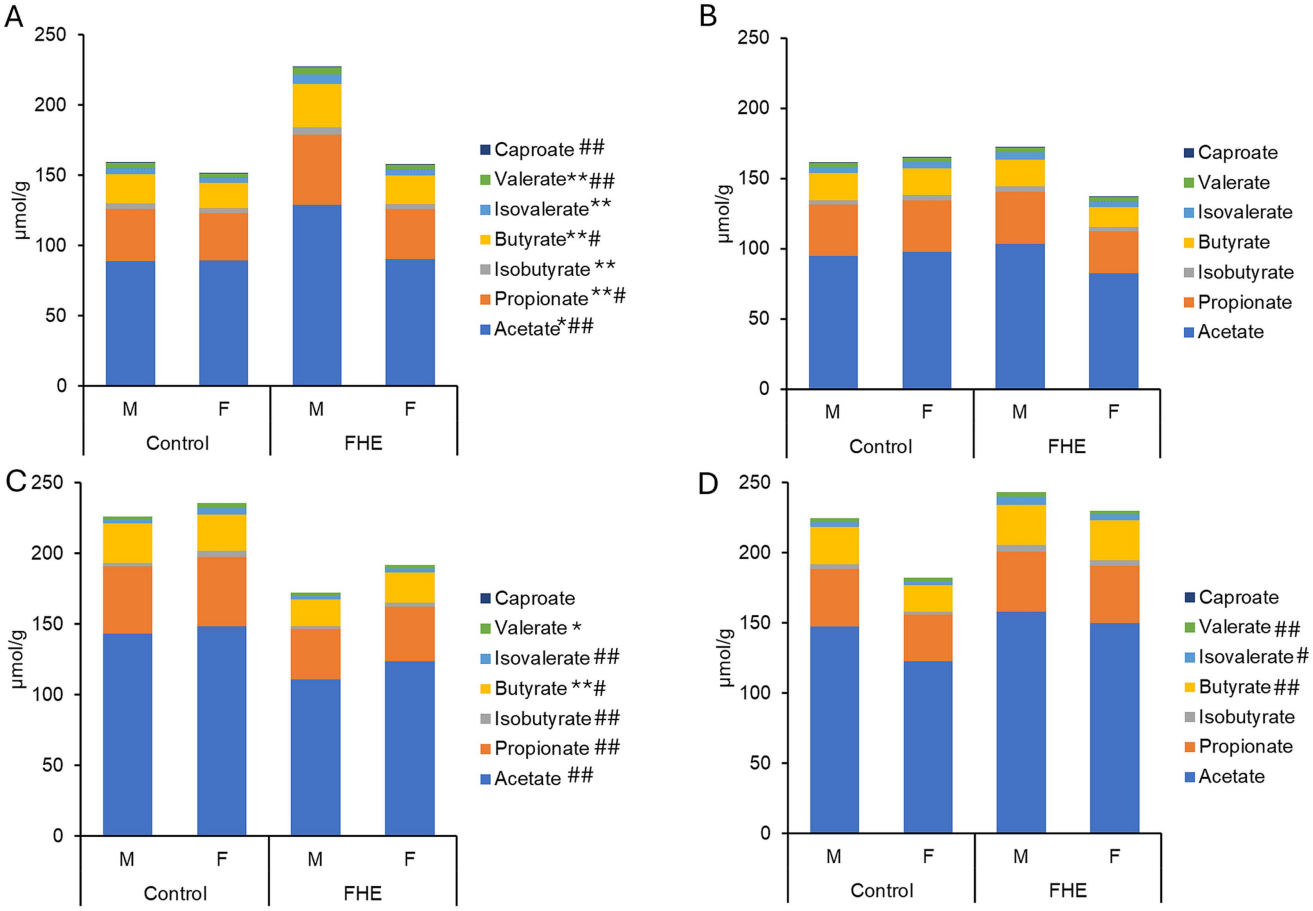
Differences in concentrations of short-chain fatty acids (μmol/g) in feces of pigs fed the control diet or diet with added fermented herbal extract (grey bar): **(A)** mid-fattening **(A)** and end of fattening period **(B)** on Farm B as well as mid-fattening **(C)** and end of fattening period **(D)** on Farm C. FHE, fermented herbal extract; F, gilt; M, barrow. Values are least-squares means ± standard error of the mean. Effect of sex: **p < 0.05; *p < 0.10; effect of diet: ## *p* < 0.05; # *p* < 0.10.

### Fecal short-chain fatty acid concentrations

On Farm A, the SCFA concentrations and molar proportions were similar between sexes and not influenced by the FHE addition in the weaner period (Figure 5; Supplementary Table S8). On Farm B, there were also no effects of sex and FHE in the weaner period; however, they were detectable in the mid-fattening period. Except for caproate, SCFA concentrations were higher in barrows than in gilts (*p* < 0.05), particularly in the FHE treatment. This was indicated by trends for sex × treatment interactions for total SCFA (*p* = 0.086) and acetate (*p* = 0.054). The observed FHE effect was therefore due to higher SCFA concentrations in the feces of barrows fed the FHE diet than in control pigs. On Farm C, FHE addition led to a 22.5% higher propionate concentration at the end of the weaner period compared to control pigs (*p* = 0.039). In the mid-fattening period, pigs fed the FHE diet on Farm C had 22.1% less total SCFA, including acetate and propionate (*p* < 0.05), and a tendency for lower butyrate (*p* = 0.093), compared with the control treatment. By the end of the fattening period, feces from pigs receiving the diet with the FHE addition tended to contain 27.1, 54.1, and 59.3% more butyrate, iso-butyrate, and iso-valerate, respectively, than the control (*p* < 0.10).

## Discussion

Different factors can influence the effects of botanicals on nutrient digestion and microbial activity in the porcine gut, which can increase the variability of results across studies but also across pig farms when applied in practice (3, 10, 13). Due to the influence of environmental factors (i.e., microbial environment in the barn and actual dietary composition) on the actual gut microbiota composition at one farm (10, 14), it can be assumed that the effects of botanicals such as FHE may vary from farm to farm. Despite this understanding, little research has been conducted on how the effect of a plant-based feed additive would differ when fed to weaned and finishing pigs on several practical farms. To investigate potential variation in effects among farms, we used a commercial product that was produced under standardized conditions. The present results support that the effects of the same FHE product on the fecal microbiota composition, their metabolites, and ATTD noticeably differed among the three farms. They underline the importance that feed additives should be tested on several farms in order to make a general statement about their effectiveness and modes of action. Three major factors may have contributed to the diverging results across farms in the current study: 1) the diet on Farms A and C: barley-corn-based diets were fed, whereas on Farm B corn-cobb-mix-based diets were fed; 2) the husbandry system: Farms A and C reared their pigs on flat decks, whereas Farm B provided a structured pen with straw bedding; and 3) the actual age of the pigs at sampling, with the biggest age difference in the weaner period. The sex of the pig influenced some parameters. However, the sex effects were not the same on the three farms. From the maturational stage of the gilts, we would have expected more similar results for sex effects in the fattening period on Farms B and C, which was not the case. To give an example, gilts seemed to use the nutrients in the feed more efficiently than the barrows at the end of the fattening period on Farm B, but not on Farm C. Instead, gilts had a higher ATTD of DM and CP compared to barrows in the mid-fattening period on Farm C but not on Farm B. Moreover, the observed sex effects on SCFA and bacterial diversity did not correspond to the influence of sex on the ATTD data. Therefore, no clear conclusion about the influence of sex on the study variables could be drawn. Lastly, regarding the interpretation of the present results, we used feces as a proxy to be able to sample each pig multiple times throughout the weaning-fattening period (24, 25). Therefore, current microbial results are representative of the distal large intestine but not of the upper gut (26). Likewise, the results for the digestibility coefficients are influenced by microbial action on feed residuals in the large intestine (16, 27) and thus do not fully reflect the FHE effects on small intestinal digestion.

Several components in the FHE may have influenced the digestive processes. Tannins from birch leaves, raspberry leaves, and yarrow in the FHE may have decreased the water content in digesta due to their astringent properties (1, 2). We observed a trend suggesting that the FHE influenced the DM content in pigs’ feces in the weaner period on Farms A and C, but the effect was opposite on the two farms. Reasons might be the dietary composition and the age of the animals, which may have interacted with the FHE effect on water retention in digesta and digesta passage. The FHE-related differences in the fecal DM content were rather small and probably not noticeable when visually inspecting the fecal consistency. Bitter components in the FHE, for instance, from birch leaves, raspberry leaves, and yarrow, may have stimulated gastric and intestinal secretions and, by that, nutrient digestion (1, 2). However, the present results for ATTD did not support this assumption. Only on Farm B, where pigs received a corn-cobb-mix-based diet, the FHE affected the CA excretion but in opposite directions in the weaner and mid-fattening period, which may be interesting to follow up on in the future to clarify the effects of the FHE on mineral absorption. As mentioned above, microbial activity in the large intestine (16, 27) may have hidden potential FHE effects when using the ATTD as a proxy for the digestibility of the diet, which may be supported by the observed changes in the bacterial community. Here, it should also be noted that we could only collect feces on 1 day per production state (age) on each farm. Slight segregation of feed particles during the transport of the mixed feed through tubes might have occurred. This may have caused small changes in the uptake of the acid-insoluble ash with the feed ingredients, which may have been better balanced if feces were collected on several days. Contrary to our assumption, fecal CP concentrations were similar between treatments; thus, we had to reject the hypothesis that the FHE reduced the fecal N excretion. Due to the single-phase feeding in the fattening period, however, an oversupply of dietary CP may have masked potential FHE effects on the N excretion in feces on Farms B and C. Also, the ATTD coefficients and fecal DM were markedly lower in fattening pigs from Farm C compared to Farm B, which may indicate a higher passage of digesta in pigs from Farm C.

The results for the beta-diversity and species richness confirmed a strong effect of the farm on the fecal bacterial communities. On all farms, we observed differences in bacterial diversity, genera abundances, and/or SCFA concentrations between treatments, supporting gut microbiota modulating capacities of the FHE, especially in the younger pigs. The biological action of the FHE on the gut microbiota may be linked to secondary compounds of the herbs but also to microbes (i.e., lactobacilli and yeast) and their metabolites produced during the fermentation process. We did not detect the *Lactobacillaceae* species used to ferment the FHE in the fecal bacterial community. A similar observation was made previously for fermented Chinese herbs, which were fermented with *Bacillus subtilis* (9). As the cultures were orally ingested, the *Lactobacillaceae* species may have been present in the stomach or upper small intestine, where *Lactobacillaceae* are generally the dominating taxa (28). Overall, it can be assumed that the majority of the observed FHE effects on the fecal microbiota were indirect effects from FHE-induced microbial changes in the upper sections of the gastrointestinal tract and changes in fermentable substrate flow caused by the effect of FHE metabolites, like bitter compounds, on digestion in the stomach and small intestine. From the herbal composition, a great number of differently acting secondary compounds (e.g., essential oils, saponins, polyphenols, and tannins) (2, 29) in the FHE acted on the gut microbiota. The fact that more FHE effects on species richness and relative bacterial abundances were observed in the weaner period than in the fattening period may be linked to the absorbability of the secondary plant metabolites and the maturational stage of the gut. We can assume that the fermentation of the plants may have rendered the saponins, tannins, and essential oils present in the FHE more absorbable from the gut, as reported for Asian fermented herbs before (30). However, the developmental stage and length of the gut may have limited their absorption in the weaner pigs.

The FHE seemed to diversify the fecal community in the weaner period on Farms A and C but not on Farm B. The age difference in the weaner period of the pigs did not seem to be the major factor for the diverging results, as indicated by the beta-diversity. This effect of the FHE addition may have been linked to the type of diets—barley-corn-based diets on Farms A and C, and corn-cobb-mix-based diet on Farm B. If we compare the substrate available in the distal large intestine, the ATTD coefficients among farms would support this assumption, which were roughly 4% lower for the ATTD of DM and CP on Farms A and C, respectively, compared to Farm B. Increased diversification in the weaner period may represent a benefit of the FHE as it confers a more resilient microbial community towards the invasion of pathogens and thus to gut homeostasis (31).

Data on the relative abundances of the predominant bacterial genera in feces also suggested that the FHE addition had a greater capacity to modulate the bacterial composition in the weaner phase compared to the mid- and end-fattening period on Farms B and C. However, FHE effects largely diverged among farms at each production stage, and as described for the diversity, it might be linked to the fermentable substrate available in the distal large intestine. Accordingly, changes in the flow of fermentable substrate due to the FHE may have provided a growth advantage to the metabolically versatile genus *Prevotella* (32) and lipolytic *Anaerovibrio* (33) in the feces of weaner pigs on Farm A. On Farm B, the drastically lowered abundance of amylolytic *Lactobacillus* (34) with the added FHE may have been indicative of lower availability of easily fermentable carbohydrates due to an FHE-related improved carbohydrate digestion or fermentation. In turn, the composition of the microbiota changed towards a higher abundance of taxa that can recycle a variety of organic molecules, including amino acids, sugars, and glycoproteins from mucin, such as *Clostridium* sensu stricto and *Turicibacter* (35). On Farm C, there were alterations in fecal abundances within the family *Prevotellaceae* and more *Lactobacillaceae* at the end of the weaner phase, which may also be indicative of changes in the availability of fermentable carbohydrates in the distal large intestine due to the FHE. As a propionate- and butyrate-producer, *Megasphaera* relies on cross-feeding relationships with lactate-producers (36). Therefore, it is thinkable that there was an increased cross-feeding of lactate by *Lactobacillaceae* to *Megasphaera* in the distal large intestine of weaner pigs fed the FHE on Farm C. Its higher abundance may explain the higher propionate concentration of pigs fed the FHE diet. Short-chain fatty acids play fundamental roles in the promotion of gut homeostasis, mucosal cell metabolism, and the regulation of inflammatory responses (37, 38). Although most benefits have been related to butyrate, the other straight-chained SCFAs possess similar capabilities (37, 38). Therefore, the stimulation of propionate fermentation in the distal large intestine may represent a benefit of the FHE for the weaner pigs. However, this FHE effect was limited to Farm C. On Farm B, a positive effect of the FHE on the fecal SCFA concentrations, including all straight-chained SCFA, was limited to the mid-fattening period and therefore to the barrows receiving the diet with the FHE addition. This observation could be an interactive effect of the FHE with the feed intake level of the barrows and thus higher substrate flow in the distal large intestine compared to the gilts (39). We did not find a similar sex effect in the FHE group for the bacterial genus abundances in feces, indicating that the microbiota was more affected at the functional level. Pigs of the same age (mid-fattening period) on Farm C did not show a similar positive effect of the FHE on SCFA concentrations compared to Farm B. One explanation may be the different basal diets on the two farms. Another explanation is related to the fact that SCFA in feces are net concentrations, which are influenced by digestion, production, absorption, and passage rate (40). It can be speculated whether there was an enhanced absorption of SCFA in the distal large intestine of pigs fed the FHE diet compared to those fed the control diet. Conversely, at the end of the fattening period, there was a beneficial effect of FHE to promote higher butyrate concentrations but also to increase branched-chain fatty acids in feces; the latter suggesting residual dietary protein or mucin fermentation in the distal large intestine. There exists ambiguous information regarding the role of branched-chain fatty acids in gut homeostasis (41, 42). However, in the most distal segment of the gut, higher branched-chain fatty acids may be more indicative of improved fermentable carbohydrate utilization in the more proximal parts of the gut (40).

## Conclusion

The present results show that the farm had a considerable impact on the effect of the FHE on our target parameters, evidencing the influence of different environments and diets on the modulatory abilities of such feed additives on the gut microbiota and digestion in weaned and fattening pigs. The present results alluded to a certain beneficial potential of the present FHE treatment to stimulate the bacterial diversification in feces of pigs when fed a barley-corn-based diet in the weaner period and to increase anti-inflammatory SCFA in feces of pigs either in the weaner or fattening period. Considering the limitation of using feces as a proxy for the occurrences in the large intestine, it may be worth evaluating the modulatory potential of the present FHE treatment on the microbial community and functionality in the upper digestive tract in future studies.

## Data Availability

The datasets generated for this study were deposited into the NCBI Bioproject databank under accession number PRJNA1223177.

## Footnotes

^1^ www.hofkultur.at
