# Supplementary material for: Potential of fermented herbal extracts to modulate digestion and gut microbiota during the weaner and fattening period on commercial pig farms
Source: Front Vet Sci. 2025 Nov 10;12:1620045. doi: 10.3389/fvets.2025.1620045 (PMC12642953; doi:10.3389/fvets.2025.1620045)
Supplement: Supplementary file 1 [file Table_1.DOCX]

Supplementary Material

**Supplementary Table S1.** Description of farms.

|  | Farm A | Farm B | Farm C |
| --- | --- | --- | --- |
| Farm characteristics | Farrow-to-finish farm  125 sows  450 fattening places | Farrow-to-finish farm  65 sows  450 fattening places | Farrow-to-finish farm  100 sows  400 fattening places |
| Feeding system | Tube chain feeding, dry  Weaner phase: Animal:feeding place ratio: 1:3  Fattening phase: Animal:feeding place ratio: 1:4 | Spotmix system, dry  Weaner phase: Animal:feeding place ratio: 1:1  Fattening phase: Animal:feeding place ratio: 1:3 | Spotmix system, dry  Weaner phase: Animal:feeding place ratio: 1:4  Fattening phase: Animal:feeding place ratio: 1:2 |
| Feeding | Weaner and fattening phase:  4-times daily between 0700 and 1900 h | Weaner and fattening phase:  0500 to 0900 h  1100 to 1400 h  1700 to 2100 h | Weaner and fattening phase:  6-times daily between 0600 and 1900 h |
| Space allowance | Weaner phase:  0.5 m^2^/piglet (until 21 kg)  Fattening phase:  (more than) double space allowance e.g. 1.6m^2^/pig (until end) | Weaner phase:  0.9 m^2^/piglet (until 30 kg)  Fattening phase:  (more than) double space allowance e.g. 1.5m^2^/pig (until end) | Weaner phase:  0.3 m^2^/piglet (until 30 kg)  Fattening phase:  (almost) double space allowance e.g. 1.2m^2^/pig (until end) |
| Husbandry system | Weaner phase:  Partially slatted floor, no structure for functional areas  Fattening phase:  Outdoor run with straw bedding  Indoors fully slatted floor | Weaner phase:  Outdoor climate stable with 3 functional areas  Lying area: box with straw and heat lamps Feeding area: deep straw bedding. Defecation/drinking area: slatted floor  Fattening phase:  Outdoor run with straw bedding  Indoors fully slatted floor | Weaner phase:  Partially slatted floor, no structure for functional areas  Fattening phase:  Indoor area with straw bedding  Outdoors partially slatted floor |
| Number of pens/replicate batch | Weaner phase:  2 FHE + 2 Control = 4  Fattening phase:  1 FHE + 1 Control = 2 | Weaner phase:  2 FHE + 2 Control = 4  (except replicate batch 3: 1 FHE + 1 control = 2)  Fattening phase:  1 FHE + 1 Control = 2 | Weaner phase:  2 FHE + 2 Control = 4  Fattening phase:  2 FHE + 2 Control = 4 |
| Total number of pens | Weaner phase: 12  Fattening phase: 2 | Weaner phase: 10  Fattening phase: 6 | Weaner phase: 12  Fattening phase: 12 |
| Pigs per pen | Weaner phase: 20  Fattening phase: 40 | Weaner phase: 20-25  Fattening phase: 40-50 | Weaner phase: 20  Fattening phase: 20 |
| Total number of pigs used in experiment | n = 240 (80/replicate batch) | n = 280 (90, 90 and 100 pigs/replicate batch) | n = 240 (80 pigs/replicate batch) |

**Supplementary Table S2.** Ingredient and chemical composition of starter and fattening diets fed at the time point of fecal collections.

| Farm A |  |  | Farm B |  |  |  | Farm C |  |  |
| --- | --- | --- | --- | --- | --- | --- | --- | --- | --- |
| Ingredient (%) | Starter diet |  | Ingredient (%) | Starter diet | Fattening diet |  | Ingredient (%) | Starter diet | Fattening diet |
| Barley | 39.0 |  | Corn-Cobb-Mix | 44.0 | 50.0 |  | Barley | 24.4 | 54.0 |
| Corn | 31.7 |  | Barley | 16.8 | 12.5 |  | Corn | 26.2 | 25.0 |
| Soybean meal HP | 15.0 |  | Wheat | 5.6 | 10.0 |  | Wheat | 22.9 | - |
| Premix Pig 10^1^ | 9.2 |  | Oat | 2.8 | 2.5 |  | Soybean meal 44 | 19.9 | 18.0 |
| Protein plus^1^ | 2.5 |  | Corn | 2.8 | 0 |  | Schaumann Fiber concentrate^3^ | 2.5 | - |
| Fiber supplement^1^ | 2.3 |  | Solan 135A GVO-free^2^ | 28.0 | 25.0 |  | Schaumaphos F-VM 90 ATG^3^ | 4.2 | - |
| Oil | 0.3 |  |  |  |  |  | Schaumaphos M 70/4000^3^ | - | 3.0 |
|  |  |  |  |  |  |  |  |  |  |
| Chemical composition |  |  | Chemical composition |  |  |  | Chemical composition |  |  |
| Dry matter (g/kg) | 891 |  | Dry matter (g/kg) | 804 | 768 |  | Dry matter (g/kg) | 891.5 | 755 |
| g/kg (dry matter basis) |  |  | g/kg (dry matter basis) |  |  |  | g/kg (dry matter basis) |  |  |
| Crude protein | 180 |  | Crude protein | 179 | 175 |  | Crude protein | 178 | 180 |
| Crude fiber | 44.5 |  | Crude fiber | 39 | 42.5 |  | Crude fiber | 60.0 | 52.5 |
| Ether extract | 51.0 |  | Ether extract | 35.5 | 38.0 |  | Ether extract | 31.0 | 30.5 |
| Crude ash | 58.0 |  | Crude ash | 55.5 | 47.5 |  | Crude ash | 55.5 | 54.5 |
| Nitrogen-free extracts | 666 |  | Nitrogen-free extracts | 691 | 698 |  | Nitrogen-free extracts | 675 | 682 |
| Starch | 479 |  | Starch | 528 | 526 |  | Starch | 513 | 515 |
| Sugar | 66.0 |  | Sugar | 35.0 | 35.5 |  | Sugar | 42.5 | 37.5 |
| Metabolizable energy, MJ ME/kg (dry matter) | 15.16 |  | Metabolizable energy, MJ ME/kg (dry matter) | 15.18 | 15.21 |  | Metabolizable energy, MJ ME/kg (dry matter) | 14.33 | 14.57 |

DM, dry matter.

^1^Edlinger GmbH, Aschbach, Austria.

^2^Solan, SOLAN Kraftfutterwerk Schmalwieser GesmbH & Co KG, Bachmanning, Austria.

^3^H.Wilhelm SCHAUMANN GmbH & Co KG, Brunn am Gebirge, Austria.

**Supplementary Table S3.** Permutational multivariate analysis of variance (PERMANOVA) results for dissimilarities in the fecal bacterial communities due to production stage, treatment and sex of weaned and fattening pigs at the three farms.

| Source of variation | df | Sum of squares | R^2^ | F | *P*-value |
| --- | --- | --- | --- | --- | --- |
| Farm A | | | | | |
| Treatment | 1 | 0.08 | 0.015 | 0.523 | 0.908 |
| Sex | 1 | 0.10 | 0.020 | 0.686 | 0.785 |
| Treatment × sex | 1 | 0.21 | 0.043 | 1.431 | 0.140 |
| Residual | 31 | 4.46 | 0.922 |  |  |
| Total | 34 | 4.84 | 1.000 |  |  |
| Farm B | | | | | |
| Production stage × treatment | 5 | 3.19 | 0.284 | 5.048 | 0.001 |
| Sex | 1 | 0.22 | 0.020 | 1.749 | 0.096 |
| Production stage × treatment × sex | 5 | 0.49 | 0.044 | 0.782 | 0.804 |
| Residual | 58 | 7.33 | 0.652 |  |  |
| Total | 69 | 11.23 | 1.000 |  |  |
| Farm C | | | | | |
| Production stage × treatment | 5 | 3.18 | 0.399 | 7.810 | 0.001 |
| Sex | 1 | 0.10 | 0.013 | 1.243 | 0.232 |
| Production stage × treatment × sex | 5 | 0.36 | 0.046 | 0.895 | 0.586 |
| Residual | 53 | 4.32 | 0.542 |  |  |
| Total | 64 | 7.96 | 1.000 |  |  |

The analysis based on pairwise distance of a multivariate data set and values were obtained using type III sums of squares with 999 permutations of residuals, considering significant difference at *P* < 0.05; df, degrees of freedom; F, F-value by permutation.

^1^Production stage: weaner period, mid fattening period, and end of fattening period.

**Supplementary Table S4.** Gene copy numbers of total bacteria in feces of pigs fed the control or FHE diet at the three farms

| Total bacteria | Control diet | | FHE diet | |  | *P*-value | | |
| --- | --- | --- | --- | --- | --- | --- | --- | --- |
| (log_10_ gene copies/g feces) | M | F | M | F | SEM | Sex | Treatment | Treatment  × sex |
| Farm A |  |  |  |  |  |  |  |  |
| Weaner period | 11.0 | 11.0 | 11.0 | 10.9 | 0.033 | 0.653 | 0.525 | 0.418 |
| Farm B |  |  |  |  |  |  |  |  |
| Weaner period | 10.8 | 10.9 | 10.9 | 11.0 | 0.045 | 0.034 | 0.149 | 0.355 |
| Mid fattening period | 10.9 | 10.9 | 10.9 | 10.9 | 0.049 | 0.391 | 0.529 | 0.508 |
| End of fattening period | 11.0 | 11.0 | 11.0 | 10.9 | 0.043 | 0.518 | 0.055 | 0.589 |
| Farm C |  |  |  |  |  |  |  |  |
| Weaner period | 11.0 | 10.9 | 10.8 | 10.9 | 0.063 | 0.744 | 0.030 | 0.189 |
| Mid fattening period | 10.9 | 11.0 | 10.8 | 10.8 | 0.060 | 0.464 | 0.055 | 0.959 |
| End of fattening period | 11.1 | 10.8 | 10.9 | 10.9 | 0.069 | 0.052 | 0.744 | 0.071 |

Values are LS means ± SEM. F, gilt; FHE, fermented herbal extract; M, barrow.

**Supplementary Table S5.** Relative abundances of bacterial genera (% of total reads) in feces of pigs fed the control or FHE diet in the weaner period on Farm A.

| Phylum | Genus | Control diet | FHE diet | SEM | Treatment, *P*-value |
| --- | --- | --- | --- | --- | --- |
| F | *Terrisporobacter* | 5.49 | 5.76 | 0.969 | 0.850 |
| F | *Clostridium* sensu stricto-1 | 6.77 | 5.65 | 1.408 | 0.580 |
| B | *Prevotella-*9 | 9.53 | 11.0 | 1.583 | 0.529 |
| F | *Lactobacillus* | 11.4 | 9.61 | 1.735 | 0.463 |
| B | *Prevotellaceae* NK3B31 group | 3.14 | 2.13 | 0.758 | 0.355 |
| F | *Streptococcus* | 5.20 | 4.28 | 0.832 | 0.441 |
| F | *Oscillospiraceae* UCG-005 | 1.18 | 0.95 | 0.581 | 0.780 |
| B | *Prevotella* | 1.60 | 2.78 | 0.387 | 0.038 |
| F | *Megasphaera* | 5.68 | 5.71 | 0.903 | 0.978 |
| F | *Turicibacter* | 0.32 | 0.26 | 0.140 | 0.752 |
| F | *Blautia* | 5.39 | 5.93 | 0.715 | 0.595 |
| F | *Agathobacter* | 4.85 | 5.09 | 1.329 | 0.897 |
| F | *Lactobacillaceae* HT002 | 3.99 | 4.78 | 0.591 | 0.349 |
| B | *Alloprevotella* | 2.02 | 2.45 | 0.623 | 0.631 |
| B | *Rikenellaceae* RC9 gut group | 1.27 | 1.27 | 0.305 | 0.991 |
| F | *Ruminococcus* | 1.43 | 1.68 | 0.248 | 0.492 |
| F | *Coprococcus* | 2.17 | 2.24 | 0.622 | 0.929 |
| S | *Treponema* | 0.87 | 0.54 | 0.427 | 0.591 |
| F | *Subdoligranulum* | 3.12 | 2.33 | 0.394 | 0.170 |
| F | *Anaerovibrio* | 1.64 | 3.43 | 0.614 | 0.049 |
| F | *Phascolarctobacterium* | 1.71 | 1.59 | 0.265 | 0.738 |
| F | *Romboutsia* | 0.00 | 0.08 | 0.060 | 0.357 |
| B | *Prevotellaceae* UCG-003 | 0.86 | 0.94 | 0.289 | 0.857 |
| F | *Roseburia* | 0.56 | 1.87 | 0.759 | 0.233 |
| F | *Lachnospiraceae* XPB1014 group | 0.00 | 0.56 | 0.358 | 0.274 |
| E | *Methanobrevibacter* | 0.72 | 0.75 | 0.388 | 0.950 |
| F | *Faecalibacterium* | 1.34 | 2.30 | 0.437 | 0.130 |
| B | *Prevotella-7* | 2.73 | 1.69 | 0.716 | 0.316 |
| F | *Christensenellaceae* R-7 group | 0.25 | 0.51 | 0.261 | 0.487 |
| F | *Oscillospiraceae* UCG-002 | 1.42 | 0.73 | 0.431 | 0.265 |
| F | *Butyricicoccaceae* UCG-008 | 0.15 | 0.41 | 0.229 | 0.425 |
| F | *Eubacterium hallii* group | 0.77 | 0.91 | 0.289 | 0.732 |

Values are LS means ± SEM. FHE, fermented herbal extract. Relative abundances > 0.5% of all reads are presented. B, Bacteroidota; F, Firmicutes; S, Spirochaetota.

**Supplementary Table S6** Relative abundances of bacterial genera (% of total reads) in feces of pigs fed the control or FHE diet in the weaner and fattening period on Farm B.

|  |  | Weaner period | | | |  | Mid fattening period | | | |  | End of fattening period | | | | |
| --- | --- | --- | --- | --- | --- | --- | --- | --- | --- | --- | --- | --- | --- | --- | --- | --- |
| Phylum | Genus | Control diet | FHE diet | SEM | Treatment,  *P*-value |  | Control diet | FHE diet | SEM | Treatment,  *P*-value |  | Control diet | | FHE diet | SEM | Treatment,  *P*-value |
| F | *Terrisporobacter* | 6.10 | 6.67 | 0.895 | 0.658 |  | 10.4 | 10.9 | 1.349 | 0.779 |  | 12.7 | | 11.1 | 1.238 | 0.360 |
| F | *Clostridium* sensu stricto-1 | 2.59 | 5.42 | 1.070 | 0.077 |  | 12.1 | 9.13 | 1.833 | 0.269 |  | 14.5 | | 12.7 | 1.955 | 0.517 |
| B | *Prevotella-*9 | 9.64 | 9.63 | 1.939 | 0.995 |  | 3.72 | 4.43 | 0.956 | 0.609 |  | 1.37 | | 1.04 | 0.555 | 0.685 |
| F | *Lactobacillus* | 12.0 | 6.94 | 1.153 | 0.006 |  | 5.09 | 4.19 | 1.177 | 0.595 |  | 2.83 | | 4.40 | 1.026 | 0.292 |
| B | *Prevotellaceae* NK3B31 group | 6.50 | 4.78 | 1.154 | 0.305 |  | 6.76 | 6.20 | 1.057 | 0.710 |  | 6.90 | | 3.47 | 1.069 | 0.036 |
| F | *Streptococcus* | 0.64 | 1.63 | 0.487 | 0.168 |  | 1.37 | 2.82 | 0.678 | 0.147 |  | 4.81 | | 4.13 | 1.730 | 0.785 |
| F | *Oscillospiraceae* UCG-005 | 3.45 | 3.51 | 0.871 | 0.959 |  | 4.56 | 4.64 | 0.902 | 0.948 |  | 5.51 | | 5.34 | 0.691 | 0.859 |
| B | *Prevotella* | 2.59 | 2.60 | 0.545 | 0.989 |  | 3.82 | 2.51 | 0.930 | 0.332 |  | 1.69 | | 2.10 | 0.471 | 0.547 |
| F | *Megasphaera* | 5.09 | 3.64 | 1.125 | 0.374 |  | 1.85 | 1.26 | 0.475 | 0.389 |  | 0.37 | | 0.62 | 0.356 | 0.624 |
| F | *Turicibacter* | 0.11 | 0.92 | 0.259 | 0.040 |  | 3.42 | 4.03 | 0.889 | 0.633 |  | 4.20 | | 6.26 | 0.597 | 0.026 |
| F | *Blautia* | 4.48 | 4.09 | 1.107 | 0.806 |  | 0.36 | 2.31 | 0.659 | 0.050 |  | 0.41 | | 0.45 | 0.331 | 0.940 |
| F | *Agathobacter* | 4.17 | 2.41 | 1.126 | 0.284 |  | 1.48 | 0.00 | 0.665 | 0.132 |  | 0.02 | | 0.46 | 0.274 | 0.276 |
| F | *Lactobacillaceae* HT002 | 6.70 | 5.02 | 1.503 | 0.438 |  | 1.20 | 2.04 | 0.529 | 0.274 |  | 0.02 | | 0.57 | 0.249 | 0.136 |
| B | *Alloprevotella* | 1.49 | 2.50 | 0.365 | 0.064 |  | 2.16 | 1.80 | 0.405 | 0.543 |  | 1.86 | | 1.02 | 0.517 | 0.262 |
| B | *Rikenellaceae* RC9 gut group | 1.81 | 3.55 | 0.951 | 0.211 |  | 2.38 | 3.23 | 0.399 | 0.146 |  | 3.61 | | 4.56 | 0.765 | 0.388 |
| F | *Ruminococcus* | 2.90 | 2.27 | 0.365 | 0.241 |  | 3.57 | 3.61 | 0.626 | 0.958 |  | 3.63 | | 2.75 | 0.597 | 0.310 |
| F | *Coprococcus* | 2.75 | 2.41 | 0.570 | 0.681 |  | 4.24 | 3.50 | 1.105 | 0.644 |  | 1.13 | | 1.44 | 0.859 | 0.802 |
| S | *Treponema* | 0.39 | 1.48 | 0.498 | 0.137 |  | 2.58 | 3.26 | 0.990 | 0.631 |  | 3.58 | | 6.35 | 0.700 | 0.012 |
| F | *Subdoligranulum* | 3.10 | 2.41 | 0.550 | 0.385 |  | 0.48 | 1.19 | 0.511 | 0.337 |  | 0.24 | | 0.39 | 0.171 | 0.549 |
| F | *Anaerovibrio* | 1.76 | 1.21 | 0.362 | 0.295 |  | 1.24 | 0.70 | 0.306 | 0.221 |  | 0.40 | | 0.33 | 0.171 | 0.769 |
| F | *Phascolarctobacterium* | 1.94 | 1.88 | 0.194 | 0.815 |  | 2.07 | 1.99 | 0.180 | 0.764 |  | 1.86 | | 1.82 | 0.233 | 0.889 |
| F | *Romboutsia* | 0.00 | 0.55 | 0.307 | 0.217 |  | 0.44 | 1.46 | 0.391 | 0.080 |  | 4.52 | | 4.96 | 0.774 | 0.692 |
| B | *Prevotellaceae* UCG-003 | 0.76 | 0.90 | 0.249 | 0.693 |  | 1.84 | 1.48 | 0.384 | 0.505 |  | 1.32 | | 1.87 | 0.381 | 0.321 |
| F | *Roseburia* | 1.41 | 0.67 | 0.596 | 0.389 |  | 2.13 | 1.85 | 1.656 | 0.905 |  | 1.00 | | 1.44 | 0.842 | 0.718 |
| F | *Lachnospiraceae* XPB1014 group | 0.48 | 0.23 | 0.175 | 0.330 |  | 3.30 | 1.71 | 0.599 | 0.077 |  | 3.90 | | 2.37 | 0.655 | 0.116 |
| E | *Methanobrevibacter* | 0.44 | 1.88 | 0.677 | 0.148 |  | 1.58 | 2.69 | 0.991 | 0.437 |  | 2.39 | | 3.00 | 0.717 | 0.557 |
| F | *Faecalibacterium* | 0.64 | 1.02 | 0.495 | 0.596 |  | 0.22 | 0.19 | 0.179 | 0.914 |  | ND | ND | | - | - |
| B | *Prevotella-7* | 0.84 | 0.81 | 0.338 | 0.955 |  | 0.14 | 0.30 | 0.102 | 0.283 |  | 0.11 | | 0.16 | 0.097 | 0.733 |
| F | *Christensenellaceae* R-7 group | 0.16 | 1.63 | 0.536 | 0.068 |  | 0.60 | 1.72 | 0.552 | 0.166 |  | 1.62 | | 0.94 | 0.372 | 0.210 |
| F | *Oscillospiraceae* UCG-002 | 0.53 | 1.71 | 0.629 | 0.202 |  | 0.94 | 1.12 | 0.470 | 0.797 |  | 0.01 | | 0.25 | 0.182 | 0.378 |
| F | *Butyricicoccaceae* UCG-008 | 2.63 | 1.21 | 0.570 | 0.093 |  | 0.75 | 0.86 | 0.368 | 0.839 |  | 0.37 | | 0.29 | 0.216 | 0.791 |
| F | *Eubacterium hallii* group | 0.45 | 0.88 | 0.320 | 0.354 |  | 1.17 | 0.64 | 0.294 | 0.217 |  | 0.84 | | 0.47 | 0.243 | 0.297 |

Values are LS means ± SEM. FHE, fermented herbal extract. Relative abundances > 0.5% of all reads are presented. B, Bacteroidota; F, Firmicutes; ND, not detected; S, Spirochaetota.

**Supplementary Table S7** Relative abundances of bacterial genera (% of total reads) in feces of pigs fed the control or FHE diet in the weaner and fattening period on Farm C

|  |  | Weaner period | | | |  | Mid fattening period | | | |  | End of fattening period | | | |
| --- | --- | --- | --- | --- | --- | --- | --- | --- | --- | --- | --- | --- | --- | --- | --- |
| Phylum | Genus | Control diet | FHE diet | SEM | Treatment,  *P*-value |  | Control diet | FHE diet | SEM | Treatment,  *P*-value |  | Control diet | FHE diet | SEM | Treatment,  *P*-value |
| F | *Terrisporobacter* | 7.18 | 5.30 | 0.945 | 0.176 |  | 10.4 | 12.9 | 1.091 | 0.128 |  | 9.80 | 12.2 | 1.257 | 0.201 |
| F | *Clostridium* sensu stricto-1 | 4.84 | 4.35 | 0.813 | 0.678 |  | 9.01 | 7.83 | 0.906 | 0.372 |  | 10.0 | 11.4 | 0.974 | 0.352 |
| B | *Prevotella-*9 | 14.4 | 11.3 | 1.610 | 0.192 |  | 9.08 | 9.74 | 1.141 | 0.689 |  | 4.53 | 4.81 | 0.964 | 0.842 |
| F | *Lactobacillus* | 6.90 | 9.29 | 1.210 | 0.177 |  | 6.48 | 5.49 | 1.388 | 0.619 |  | 5.19 | 3.34 | 0.981 | 0.205 |
| B | *Prevotellaceae* NK3B31 group | 2.55 | 4.94 | 0.971 | 0.098 |  | 5.08 | 5.21 | 1.352 | 0.946 |  | 4.02 | 3.90 | 0.874 | 0.925 |
| F | *Streptococcus* | 3.63 | 5.23 | 1.048 | 0.292 |  | 5.69 | 5.18 | 1.225 | 0.770 |  | 2.08 | 3.21 | 0.624 | 0.220 |
| F | *Oscillospiraceae* UCG-005 | 0.90 | 1.08 | 0.520 | 0.806 |  | 4.14 | 4.69 | 0.639 | 0.552 |  | 7.24 | 6.28 | 0.535 | 0.226 |
| B | *Prevotella* | 5.29 | 2.74 | 0.611 | 0.008 |  | 4.16 | 6.98 | 0.799 | 0.024 |  | 6.13 | 6.46 | 0.823 | 0.781 |
| F | *Megasphaera* | 3.77 | 6.75 | 0.898 | 0.030 |  | 1.72 | 2.32 | 0.768 | 0.592 |  | 0.00 | 0.18 | 0.103 | 0.219 |
| F | *Turicibacter* | 1.38 | 0.37 | 0.288 | 0.022 |  | 5.01 | 4.08 | 0.526 | 0.229 |  | 7.63 | 8.01 | 0.824 | 0.747 |
| F | *Blautia* | 4.02 | 5.68 | 0.786 | 0.153 |  | 1.99 | 2.76 | 0.906 | 0.556 |  | 0.23 | 0.22 | 0.252 | 0.974 |
| F | *Agathobacter* | 3.87 | 3.78 | 0.839 | 0.938 |  | 2.55 | 1.44 | 1.013 | 0.453 |  | 2.10 | 1.84 | 1.355 | 0.891 |
| F | *Lactobacillaceae* HT002 | 2.09 | 3.85 | 0.477 | 0.017 |  | 1.76 | 1.18 | 0.351 | 0.261 |  | 0.29 | 0.03 | 0.136 | 0.196 |
| B | *Alloprevotella* | 4.29 | 2.02 | 0.692 | 0.032 |  | 5.12 | 3.78 | 0.699 | 0.197 |  | 3.98 | 2.41 | 0.511 | 0.048 |
| B | *Rikenellaceae* RC9 gut group | 1.37 | 0.81 | 0.265 | 0.152 |  | 2.05 | 1.89 | 0.336 | 0.733 |  | 4.19 | 3.36 | 0.349 | 0.115 |
| F | *Ruminococcus* | 1.80 | 2.37 | 0.512 | 0.443 |  | 1.94 | 1.47 | 0.392 | 0.409 |  | 2.49 | 1.54 | 0.526 | 0.223 |
| F | *Coprococcus* | 2.33 | 3.57 | 0.325 | 0.014 |  | 1.49 | 2.53 | 0.947 | 0.447 |  | 0.01 | 0.32 | 0.149 | 0.170 |
| S | *Treponema* | 0.15 | 0.69 | 0.258 | 0.161 |  | 0.49 | 0.72 | 0.240 | 0.517 |  | 2.93 | 3.35 | 0.747 | 0.701 |
| F | *Subdoligranulum* | 3.70 | 3.91 | 0.500 | 0.765 |  | 1.51 | 2.02 | 0.421 | 0.410 |  | 0.15 | 0.38 | 0.182 | 0.384 |
| F | *Anaerovibrio* | 2.54 | 3.19 | 0.513 | 0.375 |  | 2.92 | 1.71 | 0.981 | 0.395 |  | 1.31 | 1.31 | 0.274 | 0.989 |
| F | *Phascolarctobacterium* | 1.38 | 1.83 | 0.172 | 0.080 |  | 1.31 | 1.41 | 0.172 | 0.687 |  | 1.34 | 1.92 | 0.130 | 0.007 |
| F | *Romboutsia* | 0.67 | 0.00 | 0.206 | 0.032 |  | 2.62 | 1.95 | 0.302 | 0.137 |  | 3.77 | 4.08 | 0.425 | 0.616 |
| B | *Prevotellaceae* UCG-003 | 0.93 | 0.45 | 0.200 | 0.109 |  | 2.15 | 2.37 | 0.362 | 0.667 |  | 4.63 | 2.64 | 0.850 | 0.120 |
| F | *Roseburia* | 1.79 | 1.82 | 0.744 | 0.979 |  | 2.06 | 2.60 | 1.168 | 0.750 |  | 1.63 | 1.05 | 0.769 | 0.607 |
| F | *Lachnospiraceae* XPB1014 group | 0.00 | 0.37 | 0.259 | 0.330 |  | 0.69 | 0.77 | 0.339 | 0.875 |  | 1.04 | 1.93 | 0.425 | 0.161 |
| E | *Methanobrevibacter* | 0.00 | 0.09 | 0.064 | 0.330 |  | 0.00 | 0.06 | 0.039 | 0.333 |  | 0.12 | 0.08 | 0.108 | 0.820 |
| F | *Faecalibacterium* | 2.25 | 1.53 | 0.363 | 0.180 |  | 0.59 | 0.20 | 0.197 | 0.175 |  | 0 | 0.01 | 0.011 | 0.390 |
| B | *Prevotella-7* | 1.18 | 1.11 | 0.369 | 0.897 |  | 0.08 | 0.22 | 0.126 | 0.444 |  | ND | ND | - | - |
| F | *Christensenellaceae* R-7 group | 0.24 | 0.25 | 0.154 | 0.947 |  | 0.27 | 0.51 | 0.144 | 0.264 |  | 0.93 | 0.71 | 0.235 | 0.522 |
| F | *Oscillospiraceae* UCG-002 | 0.28 | 0.35 | 0.218 | 0.826 |  | 0.23 | 0.00 | 0.220 | 0.417 |  | ND | ND | - | - |
| F | *Butyricicoccaceae* UCG-008 | 0.32 | 0.23 | 0.235 | 0.801 |  | 0.18 | 0.02 | 0.119 | 0.345 |  | 0.86 | 0.34 | 0.336 | 0.294 |
| F | *Eubacterium hallii* group | 0.66 | 0.48 | 0.271 | 0.633 |  | 0.003 | 0.08 | 0.058 | 0.333 |  | 0.09 | 0.00 | 0.054 | 0.265 |

Values are LS means ± SEM. FHE, fermented herbal extract. Relative abundances > 0.5% of all reads are presented. B, Bacteroidota; F, Firmicutes; ND, not detected; S, Spirochaetota.

**Supplmentary Table S8** Differences in short-chain fatty acid concentrations (µmol/g) in feces of pigs fed the control or FHE diet in the weaner and fattening period at the three farms.

|  | Control diet | | FHE diet | |  | *P*-value | | |
| --- | --- | --- | --- | --- | --- | --- | --- | --- |
| Item | M | F | M | F | SEM | Sex | Treatment | Treatment × sex |
| Farm A |  |  |  |  |  |  |  |  |
| Weaner period |  |  |  |  |  |  |  |  |
| Total SCFA | 200.6 | 213.7 | 189.2 | 204.0 | 19.15 | 0.471 | 0.586 | 0.965 |
| Acetate | 106.3 | 106.1 | 98.5 | 107.8 | 9.07 | 0.617 | 0.740 | 0.608 |
| Propionate | 49.26 | 55.67 | 49.98 | 49.78 | 5.697 | 0.596 | 0.659 | 0.573 |
| Isobutyrate | 3.65 | 3.89 | 2.57 | 3.68 | 0.665 | 0.317 | 0.339 | 0.521 |
| Butyrate | 31.28 | 35.83 | 30.43 | 32.06 | 4.142 | 0.462 | 0.581 | 0.728 |
| Isovalerate | 4.64 | 4.96 | 3.07 | 4.88 | 0.931 | 0.270 | 0.391 | 0.437 |
| Valerate | 5.32 | 6.90 | 4.62 | 5.71 | 0.994 | 0.190 | 0.351 | 0.807 |
| Caproate | 0.15 | 0.32 | 0.00 | 0.10 | 0.148 | 0.342 | 0.202 | 0.854 |
|  |  |  |  |  |  |  |  |  |
| Farm B |  |  |  |  |  |  |  |  |
| Weaner period |  |  |  |  |  |  |  |  |
| Total SCFA | 224.3 | 190.6 | 179.9 | 170.1 | 20.51 | 0.302 | 0.131 | 0.567 |
| Acetate | 128.6 | 108.4 | 101.4 | 101.6 | 10.57 | 0.356 | 0.123 | 0.346 |
| Propionate | 51.38 | 43.83 | 41.89 | 37.46 | 5.664 | 0.304 | 0.178 | 0.758 |
| Isobutyrate | 4.00 | 3.54 | 3.20 | 3.27 | 0.397 | 0.639 | 0.195 | 0.514 |
| Butyrate | 29.22 | 25.12 | 23.27 | 20.18 | 3.304 | 0.290 | 0.116 | 0.880 |
| Isovalerate | 4.78 | 4.31 | 3.88 | 4.01 | 0.566 | 0.765 | 0.304 | 0.604 |
| Valerate | 5.52 | 4.72 | 5.73 | 3.20 | 1.273 | 0.206 | 0.615 | 0.506 |
| Caproate | 0.75 | 0.62 | 0.59 | 0.41 | 0.255 | 0.547 | 0.470 | 0.918 |
|  |  |  |  |  |  |  |  |  |
| Mid fattening period |  |  |  |  |  |  |  |  |
| Total SCFA | 159.2 | 151.6 | 227.8 | 158.1 | 17.15 | 0.036 | 0.041 | 0.086 |
| Acetate | 88.77 | 89.38 | 128.8 | 90.65 | 9.45 | 0.062 | 0.042 | 0.054 |
| Propionate | 37.37 | 33.88 | 50.45 | 35.46 | 3.97 | 0.031 | 0.080 | 0.164 |
| Isobutyrate | 3.71 | 3.17 | 4.96 | 3.41 | 0.470 | 0.038 | 0.127 | 0.296 |
| Butyrate | 20.91 | 18.09 | 31.03 | 20.61 | 3.120 | 0.047 | 0.057 | 0.238 |
| Isovalerate | 4.64 | 3.91 | 6.56 | 4.24 | 0.660 | 0.032 | 0.103 | 0.243 |
| Valerate | 3.52 | 2.89 | 5.26 | 3.19 | 0.463 | 0.009 | 0.041 | 0.135 |
| Caproate | 0.25 | 0.26 | 0.74 | 0.49 | 0.165 | 0.475 | 0.041 | 0.422 |
|  |  |  |  |  |  |  |  |  |
| End of fattening period |  |  |  |  |  |  |  |  |
| Total SCFA | 161.5 | 165.3 | 172.6 | 137.0 | 13.55 | 0.256 | 0.535 | 0.165 |
| Acetate | 95.02 | 97.75 | 103.2 | 82.46 | 7.31 | 0.234 | 0.633 | 0.128 |
| Propionate | 36.32 | 36.52 | 37.31 | 29.84 | 3.419 | 0.302 | 0.416 | 0.279 |
| Isobutyrate | 3.16 | 3.74 | 3.96 | 3.21 | 0.545 | 0.875 | 0.799 | 0.241 |
| Butyrate | 19.56 | 19.16 | 19.03 | 14.15 | 2.217 | 0.251 | 0.228 | 0.328 |
| Isovalerate | 3.94 | 4.77 | 5.11 | 4.15 | 0.768 | 0.936 | 0.724 | 0.263 |
| Valerate | 3.02 | 3.15 | 3.50 | 2.90 | 0.428 | 0.591 | 0.796 | 0.403 |
| Caproate | 0.46 | 0.22 | 0.53 | 0.30 | 0.168 | 0.184 | 0.670 | 0.973 |
|  |  |  |  |  |  |  |  |  |
| Farm C |  |  |  |  |  |  |  |  |
| Weaner period |  |  |  |  |  |  |  |  |
| Total SCFA | 182.0 | 177.3 | 191.5 | 206.0 | 15.64 | 0.756 | 0.236 | 0.548 |
| Acetate | 110.4 | 102.6 | 103.1 | 109.7 | 7.56 | 0.939 | 0.989 | 0.354 |
| Propionate | 37.98 | 38.02 | 43.40 | 49.69 | 3.859 | 0.422 | 0.039 | 0.428 |
| Isobutyrate | 2.83 | 2.37 | 2.92 | 3.53 | 0.435 | 0.855 | 0.169 | 0.234 |
| Butyrate | 23.43 | 26.69 | 33.33 | 31.61 | 4.466 | 0.865 | 0.114 | 0.584 |
| Isovalerate | 3.21 | 2.83 | 3.52 | 4.43 | 0.624 | 0.679 | 0.142 | 0.318 |
| Valerate | 4.15 | 4.43 | 5.12 | 6.66 | 0.945 | 0.348 | 0.107 | 0.519 |
| Caproate | 0.00 | 0.37 | 0.13 | 0.41 | 0.162 | 0.060 | 0.593 | 0.785 |
|  |  |  |  |  |  |  |  |  |
| Mid fattening period |  |  |  |  |  |  |  |  |
| Total SCFA | 226.3 | 235.9 | 172.2 | 192.1 | 19.81 | 0.477 | 0.025 | 0.799 |
| Acetate | 143.3 | 148.7 | 110.8 | 124.0 | 12.25 | 0.469 | 0.033 | 0.754 |
| Propionate | 47.73 | 49.06 | 35.81 | 38.43 | 3.468 | 0.586 | 0.005 | 0.856 |
| Isobutyrate | 2.46 | 4.17 | 2.03 | 2.71 | 0.683 | 0.107 | 0.183 | 0.462 |
| Butyrate | 27.70 | 25.68 | 19.11 | 21.30 | 3.644 | 0.982 | 0.093 | 0.573 |
| Isovalerate | 2.65 | 4.75 | 2.04 | 3.07 | 0.824 | 0.085 | 0.189 | 0.530 |
| Valerate | 2.43 | 3.53 | 2.45 | 2.59 | 0.496 | 0.238 | 0.360 | 0.345 |
| Caproate | ND | ND | ND | ND | - | - | - | - |
|  |  |  |  |  |  |  |  |  |
| End of fattening period |  |  |  |  |  |  |  |  |
| Total SCFA | 224.7 | 182.2 | 243.4 | 230.2 | 19.71 | 0.183 | 0.112 | 0.469 |
| Acetate | 147.8 | 122.8 | 158.1 | 149.8 | 11.81 | 0.183 | 0.135 | 0.491 |
| Propionate | 40.95 | 33.39 | 42.87 | 41.14 | 4.131 | 0.284 | 0.261 | 0.492 |
| Isobutyrate | 3.33 | 2.07 | 4.61 | 3.72 | 0.617 | 0.108 | 0.033 | 0.767 |
| Butyrate | 26.28 | 18.86 | 28.78 | 28.63 | 2.902 | 0.217 | 0.053 | 0.231 |
| Isovalerate | 3.73 | 2.34 | 5.46 | 4.22 | 0.781 | 0.117 | 0.037 | 0.927 |
| Valerate | 2.61 | 2.74 | 3.52 | 2.66 | 0.516 | 0.490 | 0.435 | 0.352 |
| Caproate | ND | ND | ND | ND | - | - | - | - |

Values are LS means ± SEM. F, gilt; FHE, fermented herbal extract; SCFA, total short-chain fatty acids; M, barrow; ND, not detected. Heptanoate was only present below detection level.
